# Magnesium in Infectious Diseases in Older People

**DOI:** 10.3390/nu13010180

**Published:** 2021-01-08

**Authors:** Ligia J. Dominguez, Nicola Veronese, Fernando Guerrero-Romero, Mario Barbagallo

**Affiliations:** 1 Geriatric Unit, Department of Internal Medicine and Geriatrics, University of Palermo, 90100 Palermo, Italy; ligia.dominguez@unipa.it (L.J.D.); mario.barbagallo@unipa.it (M.B.); 2 Mexican Institute of Social Security IMSS, Biomedical Research Unit, Durango, ZC 34067, Mexico; guerrero.romero@gmail.com

**Keywords:** magnesium, oxidative stress, inflammation, aging, infectious diseases, vitamin D, COVID-19

## Abstract

Reduced magnesium (Mg) intake is a frequent cause of deficiency with age together with reduced absorption, renal wasting, and polypharmacotherapy. Chronic Mg deficiency may result in increased oxidative stress and low-grade inflammation, which may be linked to several age-related diseases, including higher predisposition to infectious diseases. Mg might play a role in the immune response being a cofactor for immunoglobulin synthesis and other processes strictly associated with the function of T and B cells. Mg is necessary for the biosynthesis, transport, and activation of vitamin D, another key factor in the pathogenesis of infectious diseases. The regulation of cytosolic free Mg in immune cells involves Mg transport systems, such as the melastatin-like transient receptor potential 7 channel, the solute carrier family, and the magnesium transporter 1 (MAGT1). The functional importance of Mg transport in immunity was unknown until the description of the primary immunodeficiency XMEN (X-linked immunodeficiency with Mg defect, Epstein–Barr virus infection, and neoplasia) due to a genetic deficiency of MAGT1 characterized by chronic Epstein–Barr virus infection. This and other research reporting associations of Mg deficit with viral and bacterial infections indicate a possible role of Mg deficit in the recent coronavirus disease 2019 (COVID-19) and its complications. In this review, we will discuss the importance of Mg for the immune system and for infectious diseases, including the recent pandemic of COVID-19.

## 1. Introduction

About eleven thousand years ago, with the introduction of agriculture, the human beings radically modified their way of living from the remote and primitive hunter–gatherer organization towards a new form of more sedentary cohabitation, which also included the domestication of animals. The new epidemiological scenario allowed the coexistence of microorganisms, wild and domestic animals, and the human beings, which is now recognized as the origin of the most important human infectious diseases [1]. These diseases can only be sustained in large dense human populations that did not exist anywhere in the world before agriculture. Despite the fact that this story began so long ago and notwithstanding the advances in the development of vaccines and antibiotics, infectious diseases continue to be a major burden in global public health [2]. Furthermore, new emerging infectious diseases continue to be described and can lead, as at the present time, to pandemics that reveal our unpreparedness to face new infectious noxae. In fact, overcrowding and population movements can make a local infectious problem turn into a serious and feared pandemic attack in a short period of time as a consequence of globalization.

Historians increasingly recognize that infectious diseases have shaped the course of history as in the emblematic example of how more Native Americans died from microbes brought by the European conquerors rather than by their swords and guns allowing the relatively easy conquest of the new discovered territories [1]. Humanity today is again witnessing the profound changes that infectious diseases can produce in history with the tragic mortality toll and economic hardship caused by the coronavirus disease 2019 (COVID-19) pandemic.

Infections are more frequent in vulnerable populations such as older adults for a number of reasons, including the physiologic changes that accompany “normal” aging and the multimorbidity frequent in older populations with various simultaneously occurring chronic diseases as well as the medical, diagnostic, and surgical interventions that accompany them. Not only are infections more frequent in older adults, but they can be more injurious generating a cascade of complications that result in substantial human and financial costs [3].

Magnesium (Mg), a mineral of primary physiological importance, is the most abundant divalent cation in living cells. In the human body, Mg is the second most abundant intracellular cation after potassium and the fourth most common mineral in the whole body after calcium, sodium, and potassium. Mg is an essential cofactor for numerous biological processes (estimated at over 600) acting both on the enzymes as a structural or catalytic component and on the substrates [4] and it is required for oxidative phosphorylation, energy production, protein synthesis, glycolysis, and nucleic acid synthesis and stability [5,6]. This fundamental ion also plays an essential role in the active transport of other ions across cell membranes, therefore modulating neuron excitability, muscle contraction, and normal heart rhythm [7]. In the serum, Mg exists in three forms: a protein-bound fraction (25% bound to albumin and 8% bound to globulins), a chelated fraction (12%), and the metabolically active ionized fraction (55%) [5,6].

For all these reasons, Mg is a critical factor for normal cellular and body homeostasis, including the processes involving the immune system. In particular, Mg has a strong relationship with both innate and acquired immune responses, playing a key role in the signaling pathways that regulate the development, homeostasis, and activation of immune cells [8]. Mg deficiency, frequent in old age, can cause inflammation by diverse mechanisms, including activation of phagocytic cells, opening of calcium channels, activation of the N-methyl-d-aspartate (NMDA) receptor and of nuclear factor kappa-light-chain-enhancer of activated B cells (NF-κB) [9], while it can also increase oxidative stress [10,11]. The discovery of a genetic disease, X-linked immunodeficiency with magnesium defect (XMEN), that can lead to severe and chronic Epstein–Barr virus infections and neoplasia confirmed the important role of Mg as a second messenger in immunity [12,13,14].

Over the past decades, the clinical relevance and biological significance of Mg have been documented, as well as the impact of Mg on molecular and physiological processes of aging, especially those regarding the immune system and infectious diseases [11,15]. Older adults, together with the frequent magnesium deficiency due to a variety of reasons [15], undergo modifications of the immune response that can make them particularly susceptible to infections and their complications [3].

In this review, we will discuss the importance of Mg for the immune system and for infectious diseases, including the recent pandemic of COVID-19, with particular focus on older populations.

## 2. Mg and the Immune Responses

Previous and also subsequent studies have shown that Mg plays a role in the immune response as a cofactor for immunoglobulin (Ig) synthesis, C3 convertase, immune cell adherence, antibody-dependent cytolysis, IgM lymphocyte binding, macrophage response to lymphokines, and T helper–B cell adherence [8,16]. Mg reduces the expression and release of substance P and other proinflammatory molecules by controlling NF-κB activity under physiological Mg conditions and leads to increased NF-κB activation and cytokine production when in suboptimal concentrations [17]. Mg also affects acquired immunity by regulating the proliferation and development of lymphocytes [18]. Most of these studies have been carried out on experimental animals fed Mg-deficient diets. These animals also exhibited altered polymorphonuclear cell number and function together with an increased number of neutrophils, which was related to increased phagocytosis [19]. Mg deficiency also alters mast cell proliferation and function (histamine storing and secretion) and might be involved in mast cell-dependent hepatic fibrosis and steatosis [20,21]. This cation participates in human cell apoptosis, because Fas-induced B cell apoptosis is a Mg-dependent process. Elevation of intracellular free Mg concentrations are needed for Fas molecule binding expression on the B cell surface to trigger signaling pathways that cause apoptosis and cellular death [22]. Other studies confirm the importance of Mg in immunoinflammatory processes with the evidence that Mg-deficient experimental animals exhibit increased inflammation, exacerbated immune stress responses, and decreased specific immune responses [23,24,25,26].

A remarkable effect observed in Mg-deficient animals is the accelerated thymus involution even at early stages of Mg deficiency. Malpuech–Brugere et al. showed a higher level of apoptosis in thymuses from Mg-deficient rats compared to the control normally Mg-fed group starting from the second day of deficiency and accompanied by the presence of inflammatory cells. Later on, after eight days, they observed an increased proportion of epithelial reticular cells in the cortex, indicative of a remodeling process [27]. Altogether, these findings suggest that Mg deficiency can be associated with a significant impaired function in T cells.

The regulation of cytosolic free Mg in the immune cells involves Mg transporters, channels, and exchangers, such as the Mg/Na exchanger and the melastatin-like transient receptor potential 7 (TRPM7) channel [28]. TRPM7 is a non-selective Mg channel (also conducts Ca, Zn, and Na) that is expressed ubiquitously [28,29,30,31,32]. TRPM7 has a serine/threonine kinase domain, whose activity can modulate the gating of TRPM7 [33]. TRPM7 is fundamental for Mg homeostasis in immune cells. This is illustrated by the fall in cytosolic free Mg and cell cycle arrest in TRPM7-deficient B cell lines, which was partially rescued by culturing the cells in a high Mg-containing medium and by the impaired development of T cells in TRPM7 conditional knockout mice [33]. In a mouse model with a specific T cell deletion of TRPM7, T lymphocyte development was blocked at the CD4CD8 stage, resulting in decreased CD4 and CD4CD8 cells in the thymus [34]. In addition, TRPM7-deficient T cells seemed to be protected from Fas receptor-induced apoptosis [35]. Other regulators of free Mg homeostasis in immune cells include members of the solute carrier (SLC) family SLC41A1 and SLC41A2, which are homologous to the bacterial Mg transporter and expressed quite ubiquitously [29,31]. The role of SLC41A1/2 in Mg homeostasis in immune cells has been confirmed by their ectopic expression in a B cell line deficient in TRPM7, which was able to restore the reduced intracellular Mg concentrations and defective proliferation [36,37]. The third type of Mg transporter, MAGT1, is crucial in immune signaling [38,39]. With respect to the other transporters, MAGT1 is expressed at higher levels in immune and epithelial cells [40,41]. The functional importance of Mg transport in immunity was unknown until the description of a new primary immunodeficiency named XMEN (X-linked immunodeficiency with Mg defect, Epstein–Barr virus infection, and neoplasia) due to a genetic deficiency of MAGT1 [12,13,14] suggesting that Mg could function as a second messenger in cellular signaling. Patients with XMEN Patients with chronic Epstein–Barr virus infections, low CD4+ T cell counts, and defective T lymphocyte activation. These effects are hypothesized to result from a loss of phospholipase C (PLC)-g1 activation due to reduced Mg influx via MAGT1. Indeed, XMEN patients display impaired PLC signaling with reduced Ca/Mgresponses after T cell receptor stimulation and abolished expression of the natural killer activating receptor NKG2D in natural killer and CD8T cells [12,13,14]. The MAGT1-dependent Mg flux is essential for the optimal activation of PLC-g1, inositol triphosphate (IP3) generation, protein kinase Cu phosphorylation, and calcium mobilization via store-operated calcium entry [12]. MAGT1 deficiency also leads to decreased cytosolic free Mg and decreased Mg uptake in T cells and B cells [12,13]. Furthermore, it was shown that in some patients, oral Mg supplementation restored the concentration of intracellular free Mg in XMEN patients [13]. After the discovery of the XMEN disease [12,13,14], new variants of the XMEN disease have been described [42], as well as new mechanisms explaining the immunodeficiency, such as the glycosylation defects of a specific subset of N-glycoproteins and reduced killing function of cytotoxic immune cells in cells from XMEN patients [43,44].

In patients with asthma, Mg administration has been shown to promote bronchodilation and improve lung function [45,46,47]. In addition to its bronchodilating effects, one study showed that Mg supplementation was able to modulate the immune responses of acute asthmatic CD4+ T cells and decrease the secretion of type 2 CD4+ T lymphocyte cytokines [48].

## 3. Magnesium, Inflammation, and Oxidative Stress

### 3.1. Inflammation

Poor Mg diets are associated with a low-grade chronic inflammatory state, a condition associated with several chronic diseases in older people [49]. The Mg-associated low-grade chronic inflammation might be explained in in vitro studies by initiating excessive production and release of interleukin (IL)-1β and tumor necrosis factor (TNF)-α and by activating phagocytic cells, opening calcium channels, activating the NMDA receptor, and NF-κB signaling, as well as by stimulating the synthesis of nitric oxide and inflammatory markers [9,50]. Mg deficit also increases platelet aggregation and adhesiveness and inhibits growth and migration of endothelial cells, potentially altering microvascular functions [9]. Moreover, some evidence has shown that Mg concentration in acutely inflamed tissues is reduced through the activation of the IL-33/ST2 axis, further indicating the importance of Mg in inflammatory pathways [51].

In animal models, it was reported that Mg deprivation may cause several consequences that finally lead to increased inflammatory parameters and in particular to (i) marked elevation of proinflammatory molecules TNF-α, IL-1-β, IL-6, vascular cell adhesion molecules, and plasminogen activator inhibitor-1 [24]; (ii) increased number of circulating inflammatory cells [16]; and (iii) increased hepatic production and release of acute phase proteins (i.e., complement, α2-macroblobulin, fibrinogen) [9,19]. Endothelial dysfunction associated with low magnesium exposure has also been linked to the release of inflammatory mediators [52]. Conversely, magnesium sulfate supplementation was shown to mediate anti-inflammatory effects in stimulated murine macrophages via attenuation of endotoxin-induced upregulation of inflammatory mediators and NF-κB, as well as by activation of phosphoinositide 3-kinase and inhibition of L-type ion channels [53]. The calcium channel-blocking effects of Mg lead to the downstream suppression of NF-κB, IL-6, and CRP [54].

In human beings, it has been reported that low serum Mg concentrations as well as inadequate dietary Mg intake are associated with low-grade systemic inflammation [55,56,57]. Other studies have confirmed an inverse relationship between Mg intake, serum Mg, and inflammation markers [58,59,60]. Probably one of the most important contributions was made by the Women’s Health Study, in which Mg intake was found to be inversely related to systemic inflammation, as measured by serum CRP concentrations, partly justifying a higher prevalence of metabolic syndrome in those with lower Mg intake [58]. Similar results were evident using the 1999–2002 National Health and Nutrition Examination Survey (NHANES) databases [55]. Of interest, in the study by King et al. conducted in 70% of the NHANES population not taking supplements, Mg intake below the recommended daily allowance (RDA) was significantly associated with elevated CRP [55]. Recently, a study performed in a large Finish population confirmed once more the inverse relationship between low dietary magnesium intake and serum hs-CRP concentrations [60]. A meta-analysis of eight randomized controlled trials (RCTs) evaluating the impact of Mg supplementation on CRP found a significant reduction in serum CRP concentrations following Mg supplementation, which was independent of the dosage of Mg supplementation or the duration of follow-up. [61]. Nevertheless, these results in small RCTs should be confirmed by larger and longer future investigations.

### 3.2. Oxidative Stress

Mg deficiency has been associated with increased oxidative stress and decreased antioxidant defense barriers. Previous in vitro studies have shown that Mg deficiency results in an increased production of oxygen-derived free radicals in various tissues [9,10], increased free radical-elicited oxidative tissue damage [10], increased production of superoxide anion by inflammatory cells [62], decreased antioxidant enzyme expression and activity [63], decreased cellular and tissue antioxidant concentrations [63], and increased oxygen peroxide production [64].

In animal models, Mg deficiency has been shown to increase lipid peroxidation and malondialdehyde and to decrease hepatic glutathione, superoxide dismutase, and vitamin E [65]; therefore, increasing oxidative stress concentrations. In this regard, our group has suggested an association between the action of Mg deficit in altering the antioxidant capacity and in activating oxidative stress, inflammation, and lipid oxidation that justify a high presence of metabolic conditions in people with low Mg intake or low serum Mg concentrations [7]. Mg itself seems to have antioxidant properties scavenging oxygen radicals, possibly by affecting the rate of spontaneous dismutation of the superoxide ion [64].

It has been shown that low serum Mg concentrations can stimulate Mg transporters such as TRPM7 and SLC41A [66], provoking the outflow of Mg from cells in order to increase serum Mg concentrations. As a consequence, intracellular Mg concentrations may decrease leading to modifications in cellular signaling functions depending on Mg and ATP. The reduction of intracellular Mg may elicit Mg stores in the mitochondria to release Mg [67] through SLC41A3 [68]. This drop in mitochondrial Mg content may further alter Mg- and ATP-linked mitochondrial signaling and functions, which may help explain the mitochondrial overproduction of free radicals, also called reactive oxygen species (ROS), and the reduction in ATP observed in Mg-deficient animal models [69,70].

Recently, it was shown that diabetic mice with Mg deficiency had increased mitochondrial oxidative stress, which contributed to cardiac diastolic dysfunction reversed after Mg supplementation [69]. This confirms that Mg can act as a mitochondrial antioxidant. According to a number of experimental studies, Mg deficiency disrupts mitochondrial function by diverse mechanisms, including alterations in coupled respiration [71,72,73], increased mitochondrial ROS production [9,10,69,70,74], suppression of the antioxidant defense system (e.g., superoxide dismutase, glutathione, catalase, vitamin E) [63,64,65,75,76,77], induction of calcium overload via the mitochondrial calcium uniporter [69,78,79], attenuation of pro-survival signaling [80,81,82], as well as by promoting the opening of the mitochondrial ATP-sensitive potassium channel [83], the inner membrane anion channel [84], and the mitochondrial permeability transition pore [85]. All these actions lead to the depolarization of the mitochondrial membrane potential [78]. Contrariwise, there are studies showing that Mg repletion improves mitochondrial function by diverse mechanisms, including the suppression of mitochondrial ROS overproduction [69,70], inhibition of the mitochondrial permeability transition pore opening and cytochrome C release [86,87,88], preservation of the mitochondrial membrane potential [89,90], reduction of the mitochondrial calcium accumulation [91,92,93], increase in protein expression of the anti-apoptotic B cell lymphoma 2 (Bcl-2) family and concurrent decrease of pro-apoptotic protein expression (such as of the Bcl-2-associated X protein) [80,90], decreasing apoptosis by quenching the activation of hypoxia-inducible factor 1-alpha and p38 mitogen-activated protein kinase/c-Jun N-terminal kinase (p38/JNK) signaling [90], and downregulating autophagy [93].

As mentioned, aging is characterized by a chronic low-grade inflammatory state that involves several tissues and organs frequently associated with multiple chronic diseases, and that has been named “inflammaging” [49]. Franceschi et al. [94] propose that the major source of inflammatory stimuli and oxidative stress is represented by endogenous/self, misplaced, or altered molecules resulting from damaged and/or dead cells and organelles (cell debris) recognized by receptors of the innate immune system, which are increased in old age due to a progressive decline in their disposal by the proteasome via autophagy and/or mitophagy.

Given all the abovementioned background, we proposed that the Mg deficiency, through its role in facilitating an impairment of the redox status and low-grade inflammation, might be considered a link to several age-related diseases and/or accelerated aging including a major predisposition to infectious diseases [11,15].

## 4. Mg and Vitamin D in Infectious Diseases

The study of vitamin/hormone D has undergone an enormous boost in the past decade, while its role as a hormone has been confirmed in various enzymatic, metabolic, physiological, and pathophysiological processes related to many organs and systems of the human body [95]. This growing interest is mostly due to the evidence that modest to severe vitamin D deficiency is widely prevalent around the world [96]. There is extensive agreement that an optimal vitamin D status is necessary not only for bone and muscle, but also for general health due to its association with multiple disorders including infectious diseases, primarily respiratory infections [95,96,97]. There is convincing evidence that vitamin D is an immunomodulatory hormone with significant biologic effects on the innate and adaptive immune systems [97].

### 4.1. Interaction between Mg and Vitamin D

**Figure 1:**
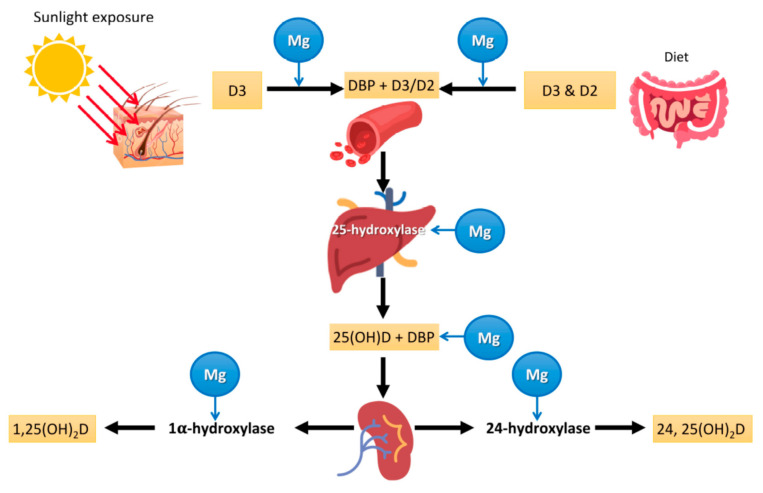
Mg and vitamin D metabolism. Vitamin D3 is produced in the skin through the action of UVB radiation reaching 7-dehydrocholesterol in the skin, followed by a thermal reaction. That vitamin D3 or oral vitamin D (D2 (ergocholecalciferol) or D3 (cholecalciferol) are converted to 25(OH)D in the liver and then to the active hormonal metabolite 1,25(OH)_2_D (calcitriol) in the kidneys or other organs as needed. As shown in the graph, Mg is a cofactor that is required for the binding of vitamin D to its transport protein, for the conversion of vitamin D by hepatic 25-hydroxlation, for the transport of 25(OH)D, and for renal 1α-hydroxylation into the active hormonal form. Therefore, all these steps are Mg-dependent. DBP: vitamin D-binding protein.

The overall metabolism and effects of vitamin D in numerous organs are well known [96]. Several steps in the metabolism of vitamin D, such as the binding of vitamin D and 25-hydroxyvitamin D —25(OH)D or calcifediol— to their transport protein and the conversion of vitamin D into the active hormonal form 1,25-dihydroxyvitamin D (calcitriol) by hepatic and renal hydroxylation, depend on Mg as a cofactor [98,99,100,101,102]; therefore, in the presence of Mg deficit, these actions would be blunted (Figure 1). Magnesium also plays a critical role in the synthesis and metabolism of parathyroid hormone (PTH), hence Mg deficiency inhibits PTH secretion or synthesis [103,104,105,106]. Mg-depleted patients with hypocalcemia despite high PTH levels suggest bone and kidney resistance to PTH [107]. Mg deficit-related hypocalcemia secondary to peripheral PTH resistance or decreased PTH secretion is further complicated by the loss of PTH stimulation of renal 1-alpha-hydroxylation with worsening vitamin D deficit [98]. Mg deficiency leading to reduced calcitriol and impaired PTH response [98] has been implicated in “Mg-dependent vitamin D-resistant rickets” [99,108]. Two studies in patients with Mg deficiency [98,109] showed that Mg infusion alone resulted in a non-significant increase in calcitriol and in 25(OH)D [98], while Mg infusion added to oral vitamin D markedly increased both serum calcitriol and 25(OH)D [109], confirming the interaction between Mg and vitamin D. These findings should be tested in larger clinical trials. Of note, vitamin D, in turn, plays a key role in the metabolism of Mg both by stimulating intestinal Mg absorption and by preventing renal Mg excretion [110]. Thus, it appears that the deficit of each of these compounds, Mg and vitamin D, feeds the deficit of the other, which may lead to a perverse cycle with further worsening of both deficits. The combined effects of Mg and vitamin D deficiency may lead to clinically relevant outcomes, such as a higher risk of fragility fractures, particularly in women [111]. It is plausible that similar harmful effects of this detrimental combination could be observed in other major clinical outcomes, such as infections.

A study by Deng et al. [112] investigated potential interactions between Mg intake, vitamin D status, and mortality. They analyzed data from NHANES 2001 to 2006 and NHANES III reporting that 12% of participants had a severe 25(OH)D deficit (<12 ng/mL) and 30% had an insufficient level of vitamin D (12 to 20 ng/mL). High total Mg intake (dietary or supplemental) was independently associated with reduced risk of vitamin D deficit or insufficiency. They also found an inverse association of serum 25(OH)D with mortality (particularly due to cardiovascular disease and colorectal cancer) that was modified by high Mg intake (i.e., the inverse association was primarily present among those with Mg intake above the median). Thus, Mg intake alone or its interaction with vitamin D intake may contribute to vitamin D status and the association of 25(OH)D with mortality risk may be modified by the level of Mg intake. A recent nested RCT within the Personalized Prevention of Colorectal Cancer Trial tested whether Mg supplementation affects vitamin D metabolism, evaluating 180 participants in a double-blind 2 × 2 factorial RCT and measuring plasma vitamin D metabolites by liquid chromatography–mass spectrometry. The analyses showed that an optimal Mg status was related to improvement of the 25(OH)D status [113].

### 4.2. Vitamin D and Infections

In addition to its musculoskeletal actions, vitamin D seems to have an important role in infectious diseases. One of the first evidences regards the impact of vitamin D on *Mycobacterium tuberculosis* infection. In this specific condition, the crucial role played by vitamin D in the immune response consists in promoting phagolysosome formation, as well as the production of the human antimicrobial peptides cathelicidin LL-37 and defensins [114,115]. The effects of vitamin D supplementation and tuberculosis have been subsequently extensively studied [116]. Furthermore, it was reported that vitamin D concentrations are associated with other infectious diseases, including acquired immune-deficiency syndrome, and respiratory diseases, particularly pneumonia [117], even if measurement of serum vitamin D concentrations may be profoundly perturbed [118]. In fact, it is still debated whether low vitamin D levels are a cause or a consequence of disease. However, it could be considered that during the course of a severe infection, the increase in energy consumption and in the demand for ATP and Mg closely related to it may decrease the efficacy of the immunomodulatory actions of vitamin D. As discussed above, low Mg can further decrease the activation of vitamin D, initiating a vicious cycle that may lead to even worse deficiencies, which are difficult to correct if they are not taken into account and detected early.

As mentioned, compelling evidence shows that vitamin D is an immunomodulatory hormone [97], while vitamin D deficit has been linked to various infective diseases, including upper respiratory and enteric infections, pneumonia, otitis media, *Clostridium* infections, vaginosis, urinary tract infections, sepsis, influenza, dengue, hepatitis B, hepatitis C, and HIV infections [119,120]. The protective properties that vitamin D exerts during infections have been attributed to upregulation of the expression of cathelicidin and beta-defensin 2 in phagocytes and epithelial cells [119]. Particular attention has been given to respiratory infections and the mechanisms of the protection given by vitamin D. This includes the maintenance of tight junctions, gap junctions, and adherens junctions, as well as induction of antiviral cytokines to interfere with the viral replicative cycle, in addition to the mentioned effects on cellular innate immunity partly through the induction of antimicrobial peptides, i.e., human cathelicidin LL-37 and defensins [120,121].

We cited the frequent association of low vitamin D status with a number of chronic diseases [95,96,97]. Intervention trials have rarely shown benefits of vitamin D supplementation as treatments or preventive measures, except for mortality in older adults [122]. Nevertheless, another important exception to the general trend is for upper respiratory tract infections: a recent meta-analysis involving 25 RCTs and data from 10,933 participants aged 0 to 95 years showed that vitamin D supplementation reduced the risk of acute respiratory tract infection among all participants. In subgroup analyses, the protective effects were better for those receiving daily or weekly doses compared to those receiving boluses and stronger for those with baseline 25(OH)D below 25 nmol/L (10 ng/mL), in whom there was a remarkable 70% lower incidence of acute respiratory infections [123].

### 4.3. Mg and Infectious Diseases

The close relationship of Mg and vitamin D and the necessity of an optimal Mg status for the synthesis, transport, and activation of vitamin D discussed in the previous subsection suggest that the higher incidence of infectious diseases associated with vitamin D deficiency can be at least in part explained by a deficit of Mg. Even if most studies regarding the direct association between poor Mg status and poor immune system function are derived from animal models (see above section on “Mg and the Immune Responses”), in human beings, Mg deficiency seems to be associated with a higher rate of infectious diseases, particularly when considering older people. As mentioned, the functional importance of Mg transport in immunity was put in evidence with the discovery of the XMEN disease that presents with defective T lymphocyte activation and chronic Epstein–Barr virus infection due to a genetic deficiency of MAGT1 and showing for the first time that Mg could function as a second messenger in cellular signaling [12,13,14]. Recent studies have added new mechanisms explaining the immunodeficiency and the development of chronic Epstein–Barr virus infection [43,44].

It has been suggested that Mg deficiency may play a role in liver diseases, especially in their progression due to a disruption in mitochondrial function, defective protein C translocation, inflammatory responses, oxidative stress, or metabolic disorders [124]. Mg may play a vital role in inhibiting the progression of HBV infection to hepatocellular cancer (HCC) [125]. Once HBV infection is established, the viral regulatory protein hepatitis B virus X amplifies the transforming growth factor (TGF)-β signal, which functions as a tumor promoter enhancing cancer metastasis and invasion by HCC. Mg administration can increase the expression of protein phosphatase Mg-dependent 1A, blocking TGF-β signaling by dephosphorylating p-Smad2/3 and thus preventing the transcription of specific genes needed for HCC growth [125].

A recent study showed that altered Mg status seems to have a prognostic role in older people affected by bacterial pneumonia. Of interest, hypomagnesemia and hypermagnesemia were both associated with excessive short-term mortality, 18.4% and 50%, respectively, compared to normal values of serum Mg [126]. Moreover, low serum Mg status was a significant predictor of frequent readmissions for acute exacerbation of chronic obstructive pulmonary disease (COPD) in a retrospective study of older adults [127].

## 5. Infectious Diseases in Old Age

Infections are a common cause of increased morbidity and mortality in older adults due to the various physiological modifications and progressive deterioration of homeostatic mechanisms, which lead to organ alterations, functional decline, multimorbidity, frailty, disability, and associated medical interventions [128], as well as to alterations in the immune response with aging [129]. Infectious diseases in older adults are usually more injurious than in younger populations and frequently generate a series of complications that result in substantial human and financial burden [3]. This is particularly true for residents in long term care facilities [130].

Data from the first-listed infectious disease (ID) hospitalizations in the USA using the Nationwide Inpatient Sample for 1998–2006 indicated that the mortality caused by acute infections was more than fifty-fold higher in persons aged over 65 years compared to that of persons aged 30–50 years [131]; older adults have a four times higher risk of being admitted to hospital for an acute infection compared to the general population [132]. In fact, infections are a frequent cause of hospitalization in older adults and hospitalization itself may lead to life-threatening nosocomial infections often caused by invasive diagnostic procedures and inappropriate use of urinary and venous catheters [133]. Older adults who survive a serious infection may afterwards have a functional deterioration that later leads to loss of self-sufficiency [134,135]. For example, pneumonia carries elevated long-term morbidity and mortality after hospitalization; over 70% of the surviving patients were reported to be readmitted to hospital at least once within the next 3 years of being hospitalized for pneumonia [136].

In the past century, there was a conspicuous decreasing trend of infectious diseases in developed countries, such as the USA, where infectious diseases went from 797:100,000 population in 1900 to 97:100,000 population in 1996 [131,137]. Conversely, taking into account only older adults, the hospital admission rate for infectious diseases increased by 13% from 1990 to 2002 [137].

The modifications in the immune system during aging, described with the term “immunosenescence” (Table 1), alter the organism’s capacity to overcome external noxae. All older adults exhibit the features of immunosenescence with variable severity; nevertheless, the degree of frailty is associated with the degree of immunocompetence [138]. As people grow old, the immune system loses the normal ability to fight infections, there is an increased susceptibility to get infections, to develop neoplasms and autoimmunity, and a reduced ability to heal skin lesions [129,139]. In general, older adults have a mild degree of immunosuppression consequent to immunosenescence in addition to the age-associated organ decline, multimorbidity, malnutrition, frailty, functional failure, geriatric syndromes, and polypharmacotherapy. All these factors together worsen the prognosis of older adults with infections [128,138]. 

Functional decline and deterioration of the immune system competence are linked to disease burden rather than chronological age. Older adults with chronic diseases (e.g., COPD, heart failure, diabetes) are more susceptible to common infections and exhibit reduced responses to vaccines when compared to those without comorbidity [140]. Thus, not all older adults exhibit hyporesponsiveness towards vaccination and some are able to maintain a fully functional immune system during old age. However, due to the deterioration of the immune response, any infection may be associated with a high risk of complications or mortality in frail older adults. For example, an influenza infection may be benign and self-limiting, but it can as well lead to complications and death or require hospital admission in a more vulnerable older patient. In the current COVID-19 pandemic, older adults and patients with pre-existing comorbidities (i.e., cardio- and cerebrovascular disease, diabetes, COPD, malignancy, chronic kidney disease, dementia) are those bearing the highest fatality rate of the disease, which is affecting the frailest groups of the population [141,142].

## 6. Magnesium and COVID-19 Pandemic

The outbreak of COVID-19 caused by severe acute respiratory syndrome coronavirus 2 (SARS-CoV2), a variant of coronavirus thought to originate in the Wuhan province in China [143], was declared a pandemic by the World Health Organization (WHO) in March 2020. According to the WHO, the confirmed cases reported worldwide at the time this review was written (14 December 2020) are over 70 million with 1,605,091 related deaths [144].

A portion of these patients develop interstitial pneumonia, which can evolve into acute respiratory distress syndrome (ARDS), requiring active hyperoxic ventilation with possible fatal outcomes [145]. COVID-19 not only affects lungs, but the virus can extend and impact profoundly many other organs and systems, including the cardiovascular system, the kidneys, the intestines, the liver, and the brain; hence, now it is considered a systemic disease. COVID-19 is characterized by a heterogeneous clinical presentation ranging from mild influenza-like symptoms to life-threatening pneumonia, cytokine storm, and multiple organ failure. Older adults are more susceptible to severe illness, to be admitted to the ICU, and to die from this disease [146,147]. This trend has been persistently present since the onset of the disease, and it is particularly high for older adults living in long-term care homes [148] due to their advanced age, poor health, multiple chronic diseases, living environment, immunosenescence, and exposure to potentially asymptomatic care providers. So far, there is no effective therapy available against COVID-19; therefore, supportive care is used currently as the mainstay of management of patient with the disease [149]. Although the mechanisms of respiratory involvement and multiple organ failure in COVID-19 are not completely clear and under investigation, cytokine storm seems to significantly contribute to the pathogenesis of the most severe manifestation of the disease [145].

Nutritional issues seem to be important in COVID-19 pathogenesis and prognosis. For example, obesity, a condition associated with low Mg intake [150,151], seems to be a negative factor for increasing mortality and hospitalizations in people affected by COVID-19 [152,153]. Moreover, as mentioned before and shown in Figure 1, Mg is a cofactor necessary for vitamin D biosynthesis, transport, and activation, while both Mg and vitamin D deficiencies have been associated with several chronic diseases. Both Mg and vitamin D deficiencies seem to be important in the pathogenesis of COVID-19 as reported by some investigations [121,154,155,156,157,158,159]. COVID-19 is associated with relevant lung [160] and cardiac impairment [161]. Again, the literature suggests that Mg plays an important role in lung and heart function [11,15,45,46,47,162]. In addition to obesity, other coexisting conditions such as hypertension [163], diabetes [164], and primarily old age [165] are associated with increased severity of COVID-19, plausibly because of an underlying chronic inflammatory state or a lower threshold for the development of organ dysfunction from the immune response. All these conditions, including old age and a chronic inflammatory state discussed above, have been associated with a low Mg status [7,11,15,166].

Patients with severe manifestations of COVID-19 may need hospitalization in intensive care units (ICU). Interestingly, up to 60% of critically ill patients in ICU have been reported to present some degree of Mg deficiency [167,168,169], predisposing these patients to serious, even life-threatening effects, also because of the consequent hypokalemia and hypocalcemia. Unfortunately, so far, no direct data regarding the importance of Mg in COVID-19 is available, probably because Mg is not measured routinely in major databases and studies [154]. In addition, serum concentrations representing only 1% of total body Mg do not accurately reflect intracellular concentrations and, finally, total body status [15]. In a thoughtful review, Wallace reported that constant monitoring of ionized Mg status with repletion, when appropriate, might be an effective strategy to influence disease contraction and progression [155]. In this regard, the literature supports several aspects of Mg as an anti-COVID-19 nutrient, including its “calcium channel-blocking” effects that lead to downstream suppression of NF-κB, IL-6, CRP [54], and other related endocrine disrupters; its role in regulating renal potassium loss; and its ability to activate and enhance the functionality of vitamin D [158], among others [155]. In a cohort observational study by Tan et al., among 43 consecutive hospitalized patients with COVID-19 aged ≥ 50 years and not requiring oxygen therapy at admission, 17 patients received a combined daily oral supplementation with 1000 IU of vitamin D3, 150 mg of Mg, and 500 mcg of vitamin B12, while 26 patients did not receive the supplementation. They found significant differences in the clinical course with fewer treated patients than controls requiring oxygen therapy (17.6 vs. 61.5%) and/or intensive care support (6 vs. 32%) during hospitalization. This small but significant study illustrates the importance of providing sufficient supplementation of these nutrients in circumstances where the requirements are most likely higher while fighting COVID-19 [156]. Other authors reviewing potential actions of Mg on SARS-Cov2 infection point toward Mg as a possible supporting treatment of COVID-19 patients, especially those critically ill and/or at highest risk of complications [154,157], including also pregnant women [157].

The hypothesis that COVID-19 pneumonia may have a vascular basis is strong [170,171]. Again, Mg has robust anti-thrombotic effects [52], while low Mg concentrations have been associated with endothelial dysfunction [172,173]. A recent systematic review and meta-analysis summarized the effects of oral Mg supplementation on vascular function in RCTs. Even if few studies were available and heterogeneity was high among the studies, in subgroup analyses, oral Mg significantly improved flow-mediated dilation in studies longer than 6 months, including unhealthy persons, persons older than 50 years, or with the BMI higher than 25 kg/m^2^ [174]. Therefore, it is possible that a chronic Mg deficiency, very frequently seen in old age [15], might create a favorable microenvironment for the virus to promote thromboembolism [154], the main feature of COVID-19.

Because no vaccinations (or definitive therapies) are available against COVID-19, we encourage specific research regarding the role of Mg in this infection, since the role of Mg in inflammation, oxidative stress, endothelial dysfunction, and immune response in infectious diseases, particularly in viral infections, is largely supported by preclinical and clinical evidence.

We discuss below some salient points intensely studied in COVID-19, which have been shown to be related to Mg in previous investigations. Below we discuss some salient points and those most studied with respect to Covid-19 and which have also been shown to be related to Mg.

They are plausible mechanisms that support the indication of maintaining an optimal Mg status to combat the severity and complications of COVID-19.

### 6.1. Cytokine Storm in COVID-19

Even if there are still many unresolved questions regarding the pathogenesis and the extreme variability in the clinical course of COVID-19, the available evidence indicate that the so called “cytokine storm”, which refers to uncontrolled overproduction of soluble markers of inflammation and which, in turn, maintains an aberrant systemic inflammatory response, is a major contributor to the occurrence of ARDS [145].

It appears that the collateral damage caused by excessive production of inflammatory mediators as the immune response attempts to clear the pathogen can be more injurious than the pathogen itself. This exuberant inflammatory response may be initially appropriate to control the infection, but if uncontrolled and continuous, the secondary multiple organ dysfunction can follow. The cascade of inflammatory mediators released during cytokine storm includes many immunoactive molecules, such as interferons, interleukins, chemokines, colony-stimulating factors, and TNF-α [175]. As we have discussed above, there is extensive evidence in experimental animals and in observational studies in humans confirming that low Mg status is associated with a chronic inflammatory state with increased levels of inflammation markers, particularly IL-6, TNF-α, and IL-33/ST2 axis (see subsection “Magnesium, Inflammation, and Oxidative Stress”). Hence, the preceding deficient Mg status associated with conditions that favor a detrimental course of COVID-19, such as aging, hypertension, and diabetes [7,11,15,166] and the Mg deficiency frequently seen in critical patients [167,168,169] may exacerbate the inflammatory response induced by SARS-CoV2, which in turn may determine increased Mg consumption resulting in further decreased intracellular levels, maintaining and propagating the uncontrolled inflammatory reaction, or cytokine storm.

**Figure 2:**
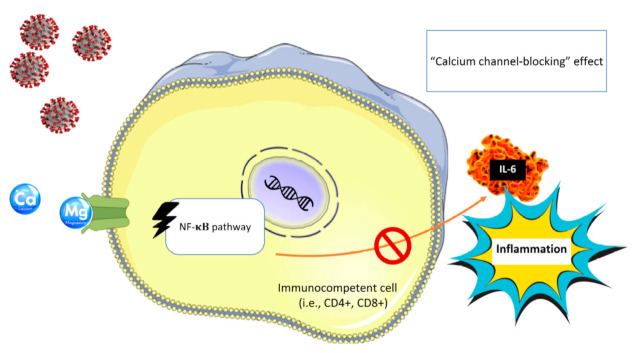
Mg’s “calcium channel-blocking” effect, which can lead to the downstream suppression of NF-κB, IL-6 and may limit systemic inflammation. NF-kB: nuclear factor 70 kappa-light-chain-enhancer of activated B cells; IL-6: interleukin 6; CD4+: T helper cell; CD8+: cytotoxic T cell.

Another well-known action of Mg relates to its calcium channel antagonist properties [176,177]. Indeed, Mg counteracts calcium as a physiological calcium blocker, similarly to synthetic calcium antagonists [178]. Interestingly, the calcium channel-blocking effects of Mg lead to the downstream suppression of NF- κB, IL-6, and CRP [54], which may limit systemic inflammation (Figure 2).

### 6.2. COVID-19 and Endothelial Dysfunction

Another pathophysiological mechanism that has been invoked and discussed extensively in the medical literature to explain the protean manifestations of COVID-19 and its multiorgan involvement is endothelial dysfunction [171]. The vascular endothelium is crucial for the maintenance of homeostasis, controlling fibrinolysis, vasomotion, inflammation, oxidative stress, vascular permeability, and structure. All these functions, acting in a concerted manner, regulate many host defense mechanisms against external noxae, but they can also contribute to disease at multiple levels when their usual homeostatic functions overreach and turn against the host, as has been reported in COVID-19 [179].

SARS-CoV-2 infects the host through the angiotensin-converting enzyme (ACE2) receptor, which is expressed in several organs, including the lung, heart, kidneys, and intestines. ACE2 receptors are also expressed by endothelial cells [179]. It is uncertain whether vascular alterations in COVID-19 are due to endothelial cell involvement by the virus. However, the endothelial cell involvement across vascular beds of different organs has been reported in a series of patients with COVID-19 [180] and anticoagulant treatment (i.e., heparin) is considered for the prevention of thromboembolic complications [181].

Mg is key for the preservation of endothelial function and vascular integrity. Low concentrations of extracellular Mg reduce endothelial cell proliferation, stimulate monocyte adhesion, and impairs vasoactive molecules, such as nitric oxide and prostacyclin [182]. Mg deficiency promotes platelet aggregation and the release of beta-thromboglobulin and thromboxanes [183]. In humans, oral Mg supplementation is significantly associated with improvement in the brachial artery’s endothelial function and exercise tolerance in patients with coronary artery disease [173] and in diabetic older adults [172]. A recent systematic review and meta-analysis reported a significantly improved flow-mediated dilation in studies with Mg supplementation for over 6 months of the follow-up, including unhealthy persons, persons older than 50 years, or with BMI > 25 kg/m^2^ [174].

### 6.3. COVID-19 and Vitamin D

**Figure 3:**
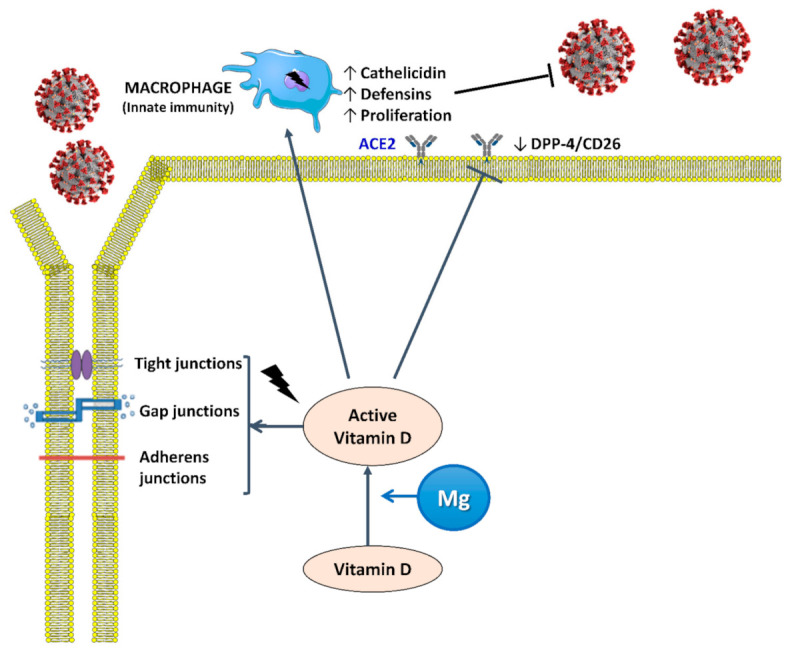
Active vitamin D (calcitriol or dihydroxycholecalciferol) helps maintain tight junctions, gap junctions, and adherens junctions in order to prevent the spread of SARS-CoV2 and induces the proliferation of macrophages and the release of cathelicidin and defensins, which are antimicrobial peptides active against a spectrum of microbes including viruses. Mg is a cofactor that is necessary for the synthesis, transport, and activation of vitamin D. ACE2: angiotensin-converting enzyme 2 receptor; DPP-4/CD26: dipeptidyl peptidase-4 receptor.

**Figure 4:**
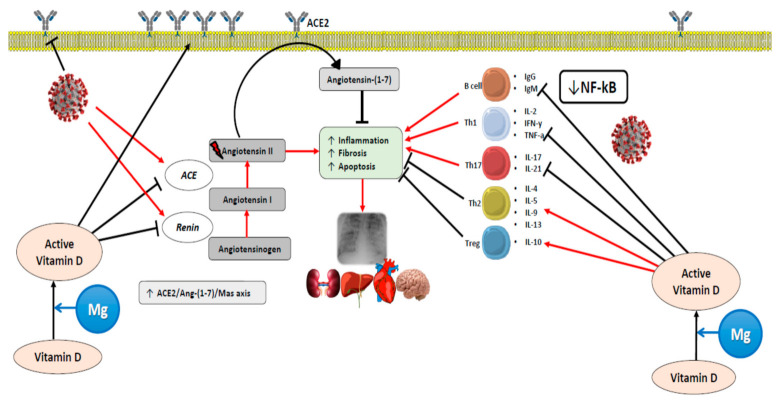
Proposed mechanisms by which vitamin D and Mg can exert actions against COVID-19. ACE, angiotensin-converting enzyme; ACE2: angiotensin-converting enzyme 2; Ang-(1-7), angiotensin (1-7); Ig, immunoglobulin; IFN-γ, interferon gamma; IL, interleukin; NF-κB, nuclear factor kappa-light-chain-enhancer of activated B cells; TNF-a, tumor necrosis factor alpha; Th, T helper cell; Treg, regulatory T cells.

As mentioned, there is convincing evidence showing that vitamin D is an immunomodulatory hormone and that its deficit has been linked to various infective diseases (see subsection “Vitamin D and Infections”). In a recent review, Grant et al. argued that vitamin D supports innate immunity, keeps the integrity of the tight junctions and the pulmonary barrier, provides immunoregulatory activity, and modulates the renin–angiotensin system, all factors of potential relevance for acute pneumonia and hyperinflammation observed in patients with COVID-19 [121](Figure 3 and Figure 4).

It is proposed that vitamin D may affect the response to COVID-19 infection by (i) supporting the production of antimicrobial peptides in the respiratory epithelium, thus making virus infection and development of COVID-19 symptoms less likely and (ii) helping to reduce the inflammatory response to SARS-CoV-2 infection. Deregulation of this response is characteristic of COVID-19 and the degree of overactivation is associated with poorer prognosis.

The medical literature on the possible actions of vitamin D in COVID-19 has exploded in recent months. Small observational studies and trials have shown encouraging results. For example, a retrospective study in a single center showed that the deficit of vitamin D was associated with increased COVID-19 risk [159], and a meta-analysis of 27 studies found that severe cases of COVID-19 more frequently had vitamin D deficiency compared with mild cases, while vitamin D insufficiency was associated with an increased possibility of hospitalization and mortality [184]. Another retrospective observational trial in 186 cases of severe COVID-19 found that low 25(OH)D levels on admission were associated with COVID-19 disease stage and mortality [185]. A study including 91 asymptomatic participants and 63 severely ill patients with COVID-19 reported a vitamin D deficiency of 33% and 97%, respectively. Serum inflammatory markers and fatality rate were higher in those with vitamin D deficiency [186]. All these results are encouraging, but it is essential to keep in mind that Mg is an indispensable cofactor for the synthesis, transport, and activation of vitamin D (Figure 1). As mentioned above, the combination of oral supplementation with Mg, vitamin D, and vitamin B12 in a sample of patients with COVID-19 showed a reduction in the need for oxygen support, intensive care hospitalization, or both [156]. Therefore, it would be advisable to correct not only vitamin D deficiency, but also Mg deficiency.

## 7. Conclusions

In this review, we reported the potential role of Mg in infectious diseases, particularly those affecting older people, a population frequently affected by deficiency of this fundamental cation. The evidence regarding the importance of Mg in these kinds of diseases is derived from animal models reporting that low-Mg diets were associated with an unfavorable profile in the immune response, oxidative stress, and inflammatory markers. These findings have been confirmed in humans by epidemiological observational studies. The discovery of XMEN, an immunodeficiency characterized by chronic Epstein–Barr virus infection, opened a particularly interesting field of research demonstrating for the first time that a cation can be a second messenger in cellular signaling and revealed the potential key role of Mg in viral infections. Mg is a cofactor for the synthesis, transport, and activation of vitamin D, which has evidence of being an important immunomodulator in several infectious diseases, including SARS-Cov2 infection responsible for the current COVID-19 pandemic. Other mechanisms described in COVID-19, such as the immune hyperresponsiveness with excessive release of inflammatory mediators leading to the cytokine storm, endothelial dysfunction, thrombotic complications, and preexisting predisposing conditions that worsen the prognosis of the COVID-19 clinical course, such as old age, diabetes, and hypertension, have all been associated with Mg deficit. Although direct data are not yet available, these concepts introduce the importance of Mg in COVID-19, a recent pandemic that is particularly harmful in older people also at higher risk for Mg deficiency and for which a definitive therapy or vaccination is still not available. It is foreseeable that an optimal Mg status might also provide healthy persons and patients that will be vaccinated against SARS-Cov2/COVID-19 with better tolerance due to the same mechanisms proposed above for possible therapeutic and disease-modulating actions.

**Figure 5:**
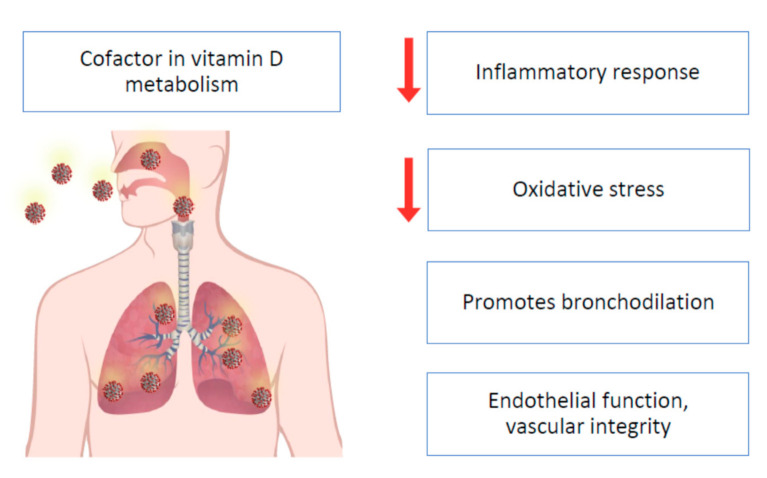
Summary of Mg’s possible effects in COVID-19.

Figure 5 summarizes the mechanisms by which maintaining an optimal Mg status may be of benefit in COVID-19.

We all are struggling to decode a new and unknown hazard which has alarmed everyone, from health providers to scientists, economists, sociologists, and rulers since December 2019. Even if Mg is not curative, it is important to ensure the correction of its deficit in order to eventually help reduce the severity of the clinical course of COVID-19. Nevertheless, further research showing the potential association between poor Mg status and COVID-19 prevalence and outcomes is consequently necessary.

## Figures and Tables

**Table 1:** Immunosenescence: modifications of the immune response with aging.

	Decreased	Increased
**Innate immune system**		
	• Anatomical and biochemical barriers: - Regeneration, sweat production, and barrier function of the skin and mucus • Hematopoietic tissue: - Total number of HSCs and hematopoietic tissue in the bone marrow, proliferative capacity of HSCs • Macrophages: - Bone marrow precursors, phagocytic capacity, and oxidative killing activity of macrophages • Neutrophils: - Chemotactic responses, migration capacity, phagocytic capacity, and superoxide generation • NK cells: - CD56^bright^ NK cell number	• NK cells: - CD56^dim^ - NK cell number
**Adaptive immune system**		
**T cells**	Thymus gland involution Number of thymic precursors Number of naïve T cellsT cell repertoire Functional activity of regulatory T cells (Treg) Number of CD4+ T cells Number of CD28+ T cells	Number of CD8+ T cells
**B cells**	B cell precursors in the bone marrow Number of B cells Plasma cell differentiation Specific antibody production B cell response to antigen exposure Diversity of the B cell repertoire Opsonizing capacity of immunglobulins	Autoreactive serum antibodies

HSCs: hematopoietic stem cells; NK: natural killer; CD: cluster of differentiation; CD4+: T helper cell; CD8+: cytotoxic T cell.

## Data Availability

No new data were created or analyzed in this study. Data sharing is not applicable to this article.
